# Influence of the Thermal Cutting Process on Cracking of Pearlitic Steels

**DOI:** 10.3390/ma14051284

**Published:** 2021-03-08

**Authors:** Lechosław Tuz, Aneta Ziewiec, Krzysztof Pańcikiewicz

**Affiliations:** Faculty of Metals Engineering and Industrial Computer Science, AGH University of Science and Technology, 30-059 Kraków, Poland; aziewiec@agh.edu.pl (A.Z.); krzysztof.pancikiewicz@agh.edu.pl (K.P.)

**Keywords:** thermal cutting, pearlitic steels, rail ways, cracking, microstructure, magnetic testing

## Abstract

The paper presents research results of the influence of heat input into high carbon rail steel during cutting processes on microstructure transformation and cracking. The massive block of steel prepared for rail rolling processes was cut and examined by nondestructive magnetic testing and destructive testing by microscopic examination and hardness measurements. The results show unfavorable microstructure changes where pearlite and transformed ledeburite were obtained. The effects of the presence of such microstructures are high hardness near to cutting surfaces (above 800 HV) and microcracks which grow into low hardness block cores during rolling and rail shaping.

## 1. Introduction

The railway system plays a vital role in world transport and is therefore an area of continuous development in terms of railway infrastructure [1,2,3,4], vehicles [5,6], management [7,8,9,10] and services [11,12,13]. The key elements of the railway infrastructure are the rails enabling trains to move, as well as frogs and turnouts enabling the change of their track. The basic materials used for these elements are hypoeutectoid or peri‑eutectoid pearlitic steels [14]. To obtain the appropriate shape, it is necessary to use casting, plastic working or heat treatment processes, enabling the profiling of the desired shape and obtaining the required mechanical properties. European standard EN 13674-1 distinguishes nine grades of steel—five grades are used in the raw state after rolling (R200, R220, R260, R260Mn, R320Cr) and four grades of rails which have been heat‑treated (R350HT, R350LHT, R370CrHT and R400HT). Their hardness can vary widely from 200 to 400 HBW [14].

The charge material for frogs or turnouts are blocks that are prepared by cutting to the technologically required size. One of the cutting processes used for this purpose is thermal cutting with an acetylene‑oxygen or a propane‑oxygen method. This process is used for cutting structural steels, but most of the alloying elements found in steels make cutting difficult and even impossible from a certain level of their content. These difficulties are mainly the result of an increase in the flowability of the liquid metal and slag, reduced heat transfer in the material and a decrease in oxidation activity. The negative effect of the alloying elements is combined, which means that in practice the carbon content must be significantly lower than the allowable one, as steels always contain other alloying elements [15].

In the thermal cutting process, a change in chemical composition occurs near the cutting surface. The surface of the metal being cut is enriched with carbon, nickel and molybdenum, while it is depleted in silicon, chromium and manganese [15,16,17,18]. A heat affected zone (HAZ) appears during cutting, but its importance is less than during welding, as the separation of the material helps to relieve some of the stresses. Structural changes in HAZ can be a problem as hardening of the edges can occur with hardenable steels, making it difficult to machine afterwards. These structural changes can influence the corrosive properties of the steels. Microcracks, similar to quenching cracks, also occur in HAZ, which can become corrosion centers and stress concentration notches [19]. Due to the presence of HAZ and geometric deviations on the edges of the cut, it is often recommended to finish them mechanically by cutting or grinding. Such operations increase the cost of execution, but even the total cost of the thermal cutting and finishing mechanical treatment is almost always much lower than mechanical cutting, which also often requires finishing.

The paper presents the results of research on cracking R260 steel used for rails and turnouts. In the production processes of shaping products, the presence of the surface microcracks, as well as macrocracks leading to fragmentation of surface fragments, was observed. Based on the analysis of manufacturing processes, it was revealed that cracks appear already at the stage of the thermal cutting process of billets into smaller parts. The paper presents the results of the tests carried out for the R260 steel, made to determine the influence of the thermal flame cutting process on the microstructural changes of the HAZ and cracking of the cutting surfaces.

## 2. Materials and Methods

**Figure 1:**
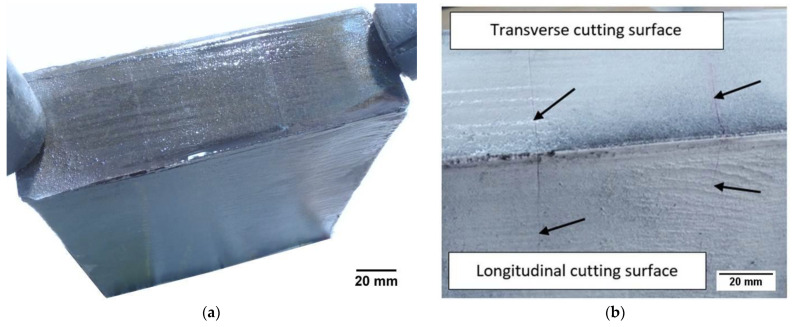
General view of the cut rod after: (**a**) cutting, (**b**) painting with primer paint and magnetic particle test.

For the tests, a rod with dimensions of 260 mm × 200 mm × 55 mm after thermal cutting with an acetylene‑oxygen burner was used. The automatic Eckert cutting machine with gas torch with A‑MD nozzle (Eckert AS, Legnica, Poland) was used to cut the workpiece. Cutting process was carried out with the following process parameters: diameter of a gas nozzle 6 mm, gas flow 600 dm^3^/min, oxygen pressure 300 kPa, cutting speed 140 mm/ min. A general view of the cut rod is presented in Figure 1a. The chemical composition of the tested steel is given in Table 1. Magnetic particle tests were performed on the cut rod. The results of the magnetic particle tests showed the cutting surfaces with cracks (Figure 1b).

Bending a sample with an existing crack was carried out on an Instron MTS testing machine (Instron, Norwood, MA, USA) at a speed of 20 mm/min, in order to open of a crack surface. The depth of the cracks was measured on the fracture using a stereoscopic microscope and it does not exceed 10 mm.

A characteristic feature of the observed cracks is the fact that the transverse and longitudinal cracks run in the same planes. Macroscopic and microscopic tests (light and scanning microscopes) and hardness measurements were performed on the samples. One sample was cut transversely to the bar axis, the other was cut along the bar length.

Macroscopic examinations were carried out using a Delta Optical XTL‑IV PRO T (Delta Optical, Mińsk Mazowiecki, Poland) stereoscopic microscope. Observation of the microstructure was performed with a Leica DM/LM (Leica, Wetzlar, Germany) light microscope. Pearlite and cementite percentages were assessed using the image analysis software Sigma Scan Pro (Version 5, 2007, Systat Software, San Jose, CA, USA). Carbon content was determined using the function (Equation (1)) based on lever rule [20].

(1) $$\%C=\frac{0.77\%\times xP}{100}+\frac{6.67\%\times xCm}{100}$$

where: *xP* is pearlite percentage, *xCm* is cementite percentage.

Fractographic studies were carried out with the use of a scanning electron microscope Phenom XL (Thermo Fisher Scientific, Waltham, MA, USA). Energy Dispersive Spectroscopy (EDS) analysis was performed at a distance of approx. 0.2, 0.3, 0.4, 1.0 and 5.0 mm from the cut surface with 20 kV and 10 nA beam for 60 s. For microscopic examination, the samples were etched in a 4% alcoholic nitric acid solution. X-Ray Diffraction (XRD) was performed on a D8 Advance Diffractometer (Bruker, Billerica, MA, USA) using Cu_Kα_ radiation. Hardness measurements were made using the Vickers method with an intender load of 10 kG (98.07 N), using a Zwick/Roell ZHU 187.5 (Zwick Roell Group, Ulm, Germany) hardness tester and with an intender load of 0.01 kG (0.09807N), using a Innovtest (Bowers Group, Bradford, UK) hardness tester.

## 3. Results and Discussion

**Figure 2:**
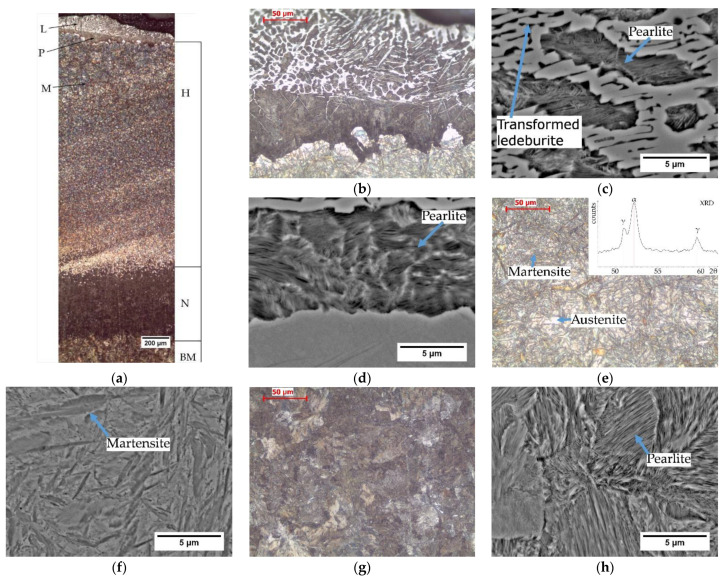
Microstructure at the cut surface: (**a**) general view (H—hardened zone, N—normalization zone, BM—base material, L— transformed ledeburite + pearlite + secondary cementite, P—pearlite with secondary cementite, M—martensite with austenite); (**b**) detail view near cut surface; (**c**) transformed ledeburite (486 HV0.01), pearlite (222 HV0.01) and secondary cementite (605 HV0.01); (**d**) pearlite with secondary cementite (375 HV0.01), (**e**,**f**) martensite with austenite (820 HV0.01) and XRD pattern in top right inset; (**g**,**h**) pearlitic microstructure of the material heated in the cutting process to a temperature lower than A_c1_—base material (227 HV0.01).

The microstructure analysis revealed the presence of a heat affected zone (HAZ) under the cutting surface. Figure 2a shows the HAZ microstructure with marked areas of hardening, normalization and the pearlitic microstructure of the base material that was not transformed during cutting.

A characteristic feature of the structure after thermal flame cutting is the presence of a layer of liquid metal with a structure containing transformed ledeburite, pearlite and secondary cementite (Figure 2b). Under the solidified metal layer, there is a dark unhardened zone with the structure of fine pearlite and secondary cementite, and then the hardened area. The presence of pearlite under the solidified metal layer is the result of a lower cooling rate of the surface layer compared to the deeper layers. The influence of heat release on the cooling rate was observed in several experiments [15,21]. The lower cooling rate is caused by the heat release in the process of solidifying the layer of nonremoved liquid and thus lowering the cooling rate of the steel under the solidified layer. The presence of cementite is the result of carbon diffusion into the steel surface layer. Further away, the cooling rate is high due to the high rate of heat dissipation to the cool material. A similar tendency was observed after the thermal cutting of S355 steel [15,21]. The further area of the material heated in the cutting process to the temperature lower than A_c1_ has a pearlitic microstructure (Figure 2g,h). The base material has a pearlitic microstructure. There is no difference between the base metal microstructure and the pearlitic microstructure of the material heated in the cutting process to the temperature lower than A_c1_.

**Figure 3:**
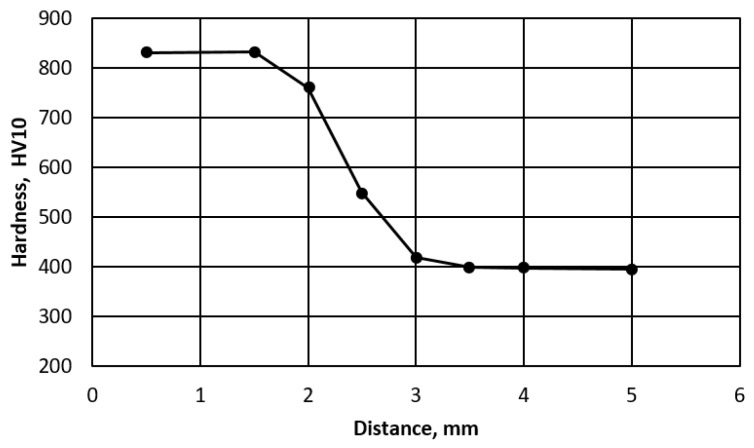
Hardness distribution from the cutting surface.

The microstructure of the hardened zone consists of martensite and a large amount of residual austenite (Figure 2e,f). The presence of austenite was confirmed by XRD (top right inset in Figure 2e). In the segregation bands with a reduced carbon and manganese content, pearlite appears at the grain boundaries. The hardened area is approx. 3 mm thick. Hardness measurements by the Vickers method showed that the hardness of the hardened layer is very high and exceeds 800 HV10 (Figure 3). It lowers as it moves away from the cut surface. Similar hardness profiles were obtained when cutting S345, S390, S700MC, S690QL steels [22,23].

**Figure 4:**
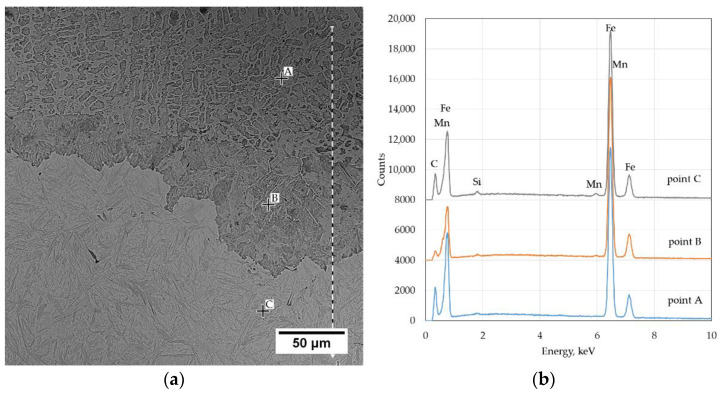
The results of the microanalysis of the chemical composition in the areas A, B, and C, performed on a SEM‑EDS: (**a**) microstructure; (**b**) SEM-EDS results

A significant problem in the thermal cutting process is the change in the chemical composition that occurs near the cut surface. The carbon content in the liquid layer was approx. 3.2% (area A in Figure 4) calculated on the basis of equation (1) [20]. The data of the volume fraction of the phases used during calculations was: 59% perlite and 41% cementite. In the nonmolten metal layer under the solidified liquid layer, the carbon content is about 1.2% (93% of pearlite and 7% of cementite) (area B in Figure 4). In the base material, the calculated carbon content was 0.77 and was close to the average carbon content in steel. The quantitative evaluation of the cementite content showed that the solidified liquid is highly carburized. 

The authors [15,16,17,18] show that carburization is the result of the selective oxidation of the steel components. The surface layer of the cutting surface is depleted in silicon, chromium, and manganese and enriched with carbon, nitrogen, nickel and molybdenum.

Surface carburization during cutting with oxygen is associated with the selective oxidation of iron. The iron from the liquid layer can easily diffuse through the liquid FeO layer to reach the cutting oxygen. Carbon does not dissolve in FeO. The direct oxidation of carbon with oxygen from the cutting stream does not take place because the rate of oxygen diffusion through the FeO layer is too slow compared to the speed of the cutting process. Much less carburization or slight decarburization of the upper part of the cutting surface is related to the direct action of the cutting oxygen on the metal near the torch nozzle. Edge carburization is possible only when a liquid FeO film is formed and carbon combustion is inhibited. The presented processes cause an intensive change in the concentration of components from the cutting surface to the base material in the area of the melted layer [15,16,17,18,23,24,25]. The present research showed that the surface layer of the cut surface is depleted of silicon and manganese in relation to the average content of these elements in steel. Figure 4 shows the photomicrograph of the cutting surface with EDS analysis sites.

The Si content at the cutting surface is 0.14% and Mn 0.3% (Table 2). This area is depleted in Si and Mn in relation to the average steel composition. A reduction in the Si content by about 70% and Mn by about 74% was observed. As you move away from the cutting surface, the content of Mn and Si increases. At a distance of 0.3 mm from the surface, it is 0.18% Si and 0.65% Mn. In the area of martensite with retained austenite, it amounts to 0.45% Si and 1.28% Mn on average, and is close to the average Si and Mn content in steel.

**Figure 5:**
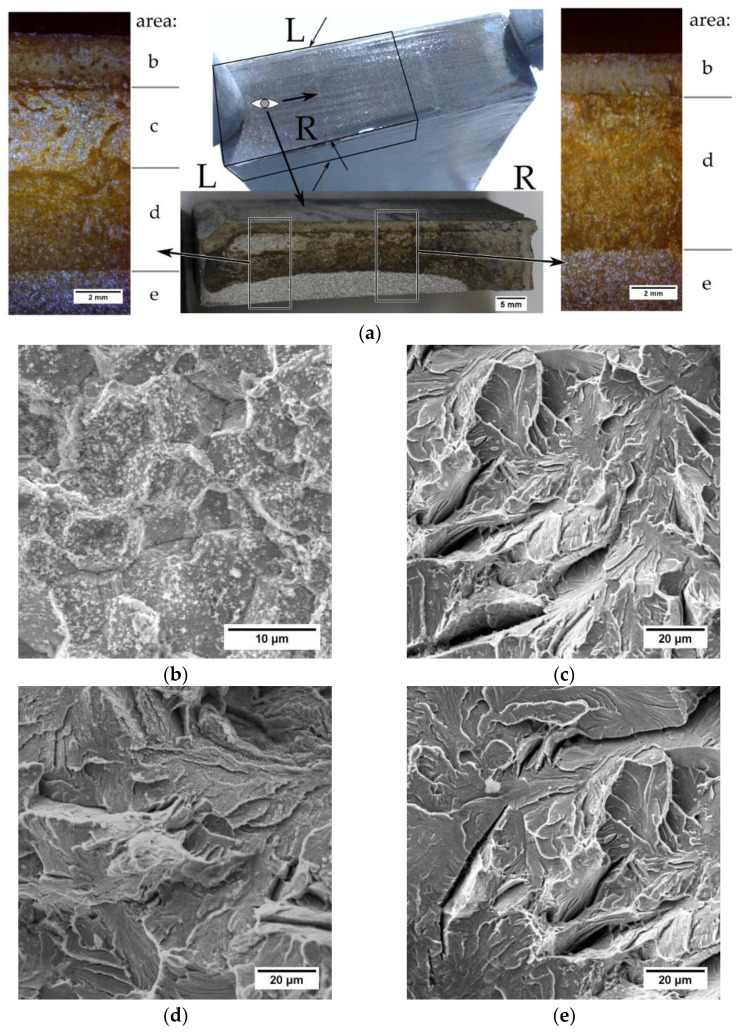
The appearance of cracks in the sample cut transversely: (**a**) general view; (**b**) brittle intercrystalline fracture in the hardened (oxidized) area; (**c**) transcrystalline brittle fracture in the pearlitic region (not oxidized); (**d**) transcrystalline fracture with visible corrosion products; (**e**) sample fracture area (not oxidized).

To determine the type of the cracks, the sample was broken from the side of the transverse cut. The analysis of the fracture surface after the sample fracture (Figure 5a, R side) showed that in the area near the surface there is a corroded intercrystalline fracture layer (area b). Under this layer, there is a transcrystalline corroded fracture area (area d). The area of breaking is brittle, transcrystalline and shows no signs of corrosion (area e). A small area of the brittle transcrystalline fracture (area c) was observed near the sheet surface. Fractographic analysis showed no signs of corrosion in this area, indicating that there was no crack (prior to the intended breaking). This area was created in the process of breaking the sample. The atypical appearance of the fracture surface (Figure 5a, L side) indicates that the fracture did not start on the surface in the hardened zone but under the hardened zone in the pearlitic structure. The resulting crack developed towards the inside of the bar and towards the hardened surface. The presence of an unoxidized atypical area (area c) near the sheet surface indicates that a crack could not have formed on the cut surface in the hardened area. Analyzing the L side in Figure 5a, it can be observed that it is not possible for a crack to arise in the hardened layer, stopped its developing and continued to nucleate at a distance from the cut surface.

**Figure 6:**
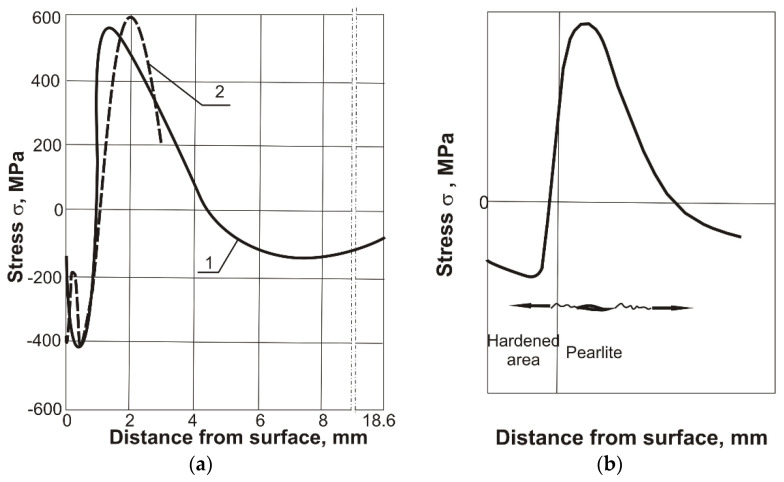
(**a**) Distributions of circumferential residual stresses in induction hardened 41Cr4 specimen to a depth of 2.3 mm (1) based on [26] and in cutting edge of 40 mm wear‑resistant steel plate (**2**) based on [19]; (**b**) scheme of crack development under the thermally cutting surface.

The cracking mechanism in the thermal cutting process can be explained by analyzing the stress distribution. Heating to high temperature in the cutting process causes the near‑surface zone to become hardened during the rapid cooling of the material. Compressive stresses occur in the hardened zone due to the phase transitions (Figure 6a) [19,26,27,28,29]. Based on the conducted research, it is possible to present the mechanism of steel cracking in the thermal cutting process. Under the hardened layer with a martensitic structure, there is an area of pearlite with maximum tensile stresses. Due to the high brittleness of the pearlite, transcrystalline brittle cracks appear in this region. Figure 6b shows a possible diagram of the development of cracks after thermal cutting. The cracks appear under the hardened layer in the area of maximum tensile stresses and during further cooling they spread both to the hardened surface and to the deeper areas of the base material.

## 4. Conclusions

The heat affected zone of R260 steel shows microstructural changes after thermal cutting. Under the cutting surface, there is a layer of solidified metal with a transformed ledeburite microstructure with secondary cementite. Under this layer there is an unhardened zone with a structure of fine pearlite and secondary cementite, and then a hardened area consisting of martensite and residual austenite. The further region has the pearlitic microstructure of the base material.

After thermal cutting in the surface layer, the chemical composition changes. There was a decrease in the content of Si by about 70%, and of Mn by about 74%. However, the carbon increases content to approx. 3.2%. In the layer of nonmelted metal under the solidified liquid layer, the carbon content is about 1.2%. As you move away from the cutting surface, the content of Mn and Si increases, approaching the average content of elements in the steel.

Based on the research and references [26,27,28,29], the mechanism of crack formation during the thermal cutting of R260 steel can be presented. The heating of the surface during thermal cutting and subsequent cooling causes phase changes to occur. Compressive stresses arise at the surface in the hardened area, and tensile stresses occur under it in the pearlitic area. High tensile stress in the pearlite region causes brittle transcrystalline cracks. These cracks spread towards the cutting surface and the base material. The depth of the cracks does not exceed 10 mm.

Due to its high C content, R260 steel has a high hardness with a surface of over 800 HV. Steels with such a high carbon content are difficult to cut. Therefore, the cutting process should be carried out on preheated bars. To minimize the possibility of cracks appearing after the cutting process, very slow cooling should be used. This will reduce the temperature gradient and stress state below the surface and prevent the excessive hardening of the surface layer.

## Figures and Tables

**Table 1:** Chemical composition of tested steel (data from the manufacturer inspection certificate 3.1).

Element	C	Mn	Si	Cr	Ni	Cu	P
Wt. %	0.79	1.17	0.48	0.10	0.09	0.18	0.018

**Table 2:** Average values of elements content in EDS analysis for the base material and areas A, B, C shown in Figure 3.

Element	Chemical Composition, wt. %			
	Area A	Area B	Area C	Base Material
Fe	99.56	99.17	98.28	98.04
Mn	0.30	0.65	1.28	1.34
Si	0.14	0.18	0.44	0.62

## Data Availability

All data is provided in full in the results section of this paper.
